# Supplementary material for: Recipient Cells Are the Source of Hematologic Malignancies After Graft Failure and Mixed Chimerism in Adults with SCD
Source: medRxiv. 2025 Jan 18:2025.01.17.25320670. Preprint. [Version 1] doi: 10.1101/2025.01.17.25320670 (PMC11759619; doi:10.1101/2025.01.17.25320670)
Supplement: Supplement 1 [file NIHPP2025.01.17.25320670v1-supplement-1.pdf]

Supplementary Table 1.Clinical characteristics of patients who developed hematologic malignancies after HCT for SCD.

Patient No.	SCD Type	Age at HCT	Sex	HCT Type	TBI Dose cGy	PT-Cy dose mg/kg	Day of Graft Failure	Dx	Time to Dx post-HCT (yr)	Cytogenetics and BM blasts at Dx	Current status
SCD-01	HbSS	35-40	Male	HLA-matched	300	0	183	MDS	2.5	Complex, <5%	Dec
SCD-02	HbSS	35-40	Male	Haplo	400	100	73	MDS	2	Complex, <5%	Dec
SCD-03	HbSS	20-25	Female	Haplo	400	100	90	AML	5.5	Complex, 20%	Dec
SCD-04	HbSS	30-35	Male	HLA-matched	300	0	74	AML	0.33	Complex, 15%-20%	Dec
SCD-05	HbSS	35-40	Female	HLA-matched	300	0	N/A	T-cell ALL	3	46XX, t(9:22)[18]/46,XY[2] BCR/ABL1 p190 fusion, 93%	Alive

HCT, hematopoietic cell transplant; SCD, sickle cell disease; HbSS, homozygous SCD; TBI, total body irradiation; PT-Cy, post-transplant cyclophosphamide; Dx; diagnosis; Haplo, haploidentical; MDS, myelodysplastic syndrome; AML, acute myeloid leukemia; ALL, acute lymphoblastic leukemia; t, translocation; Dec, deceased; N/A, not applicable.

medRxiv preprint doi: https://doi.org/10.1101/2025.01.17.25320670; this version posted January 18, 2025. The copyright holder for this preprint (which was not certified by peer review) is the author/funder, who has granted medRxiv a license to display the preprint in perpetuity.
This article is a US Government work. It is not subject to copyright under 17 USC 105 and is also made available for use under a CC0 license.

Supplementary Figure 1

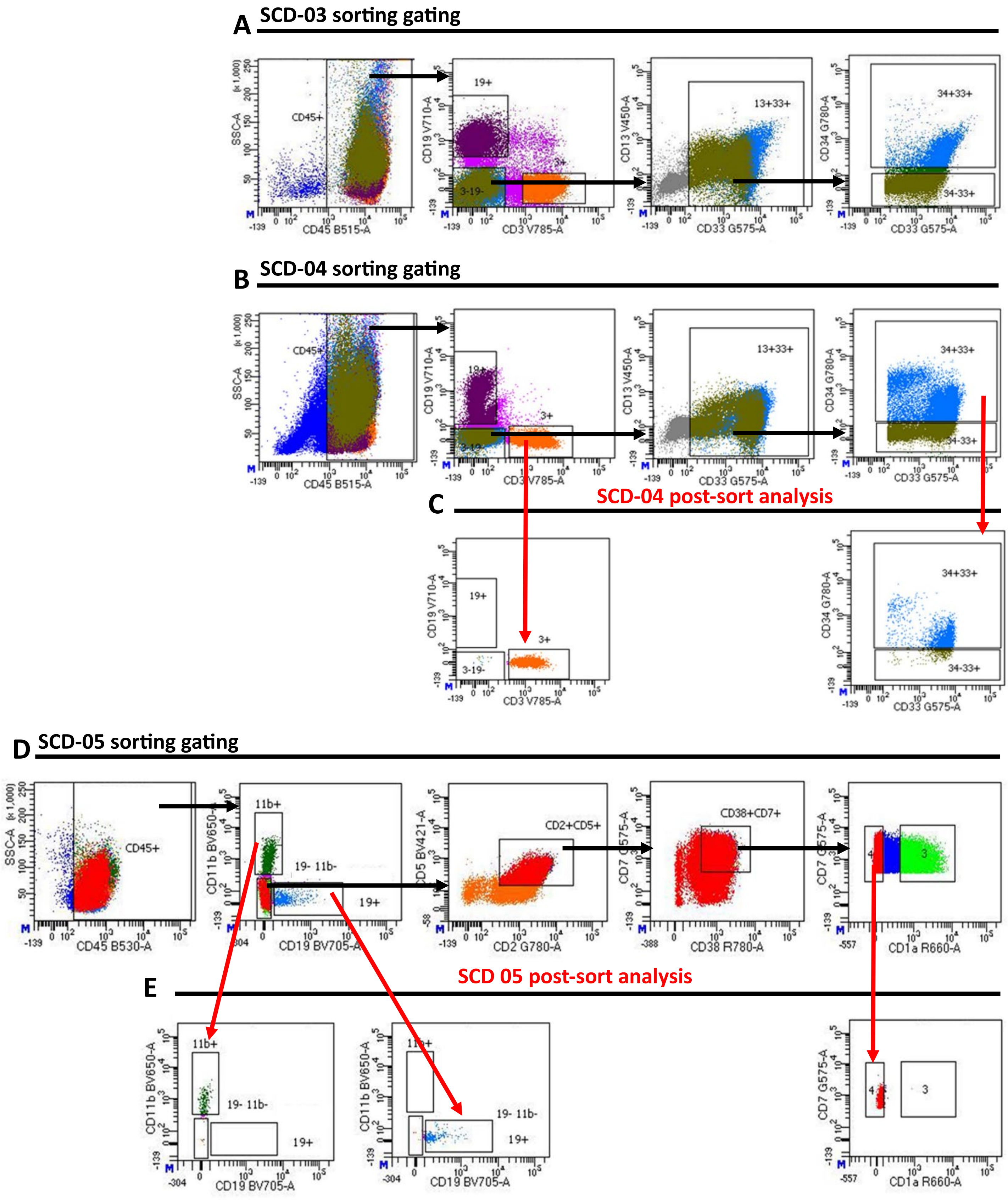

medRxiv preprint doi: https://doi.org/10.1101/2025.01.17.25320670; this version posted January 18, 2025. The copyright holder for this preprint (which was not certified by peer review) is the author/funder, who has granted medRxiv a license to display the preprint in perpetuity.
This article is a US Government work. It is not subject to copyright under 17 USC 105 and is also made available for use under a CC0 license.

Gating strategies for sorting samples with post-sort analysis. A) Gating strategy of SCD-03 PB sample. **B)** Gating strategy of SCD-04 PB sample. **C)** Post-sort analysis of SCD-04 sample showing sorted $CD3^+$ T cells (left) and $CD3^-CD19^-CD13^+CD33^+CD34^+$ cells (right). **D)** Gating strategy of SCD-05 BM sample. **E)** Post-sort analysis of SCD-05 sample showing sorted $CD11b^+$ myeloid cells (left), $CD19^+$ B cells (middle) and, $CD19^-CD11b^-CD2^+CD5^+CD7^+CD38^+CD1a^-$ cells (right). Black arrows indicate gating sequence and red arrows indicate post-sort analyses.
